# The Influence of Tea Tree Oil (*Melaleuca alternifolia*) on Fluconazole Activity against Fluconazole-Resistant *Candida albicans* Strains

**DOI:** 10.1155/2015/590470

**Published:** 2015-02-04

**Authors:** Anna Mertas, Aleksandra Garbusińska, Ewelina Szliszka, Andrzej Jureczko, Magdalena Kowalska, Wojciech Król

**Affiliations:** Department of Microbiology and Immunology, Medical University of Silesia in Katowice, Jordana 19, 41-808 Zabrze, Poland

## Abstract

The aim of this study was to evaluate the activity of fluconazole against 32 clinical strains of fluconazole-resistant *Candida albicans*, and *C. albicans* ATCC 10231 reference strain, after their exposure to sublethal concentrations of tea tree oil (TTO) or its main bioactive component terpinen-4-ol. For all tested fluconazole-resistant *C. albicans* strains TTO and terpinen-4-ol minimal inhibitory concentrations (MICs) were low, ranging from 0.06% to 0.5%. The 24-hour exposure of fluconazole-resistant *C. albicans* strains to fluconazole with sublethal dose of TTO enhanced fluconazole activity against these strains. Overall, 62.5% of isolates were classified as susceptible, 25.0% exhibited intermediate susceptibility, and 12.5% were resistant. For all of the tested clinical strains the fluconazole MIC decreased from an average of 244.0 *μ*g/mL to an average of 38.46 *μ*g/mL, and the fluconazole minimal fungicidal concentrations (MFC) decreased from an average of 254.67 *μ*g/mL to an average of 66.62 *μ*g/mL. Terpinen-4-ol was found to be more active than TTO, and strongly enhanced fluconazole activity against fluconazole-resistant *C. albicans* strains. The results of this study demonstrate that combining natural substances such as TTO and conventional drug such as fluconazole, may help treat difficult yeast infections.

## 1. Introduction

Essential oils are antiseptic substances produced by plants. Tea tree oil (TTO) is the essential oil obtained by steam distillation from the Australian native plant* Melaleuca alternifolia* and is used medicinally as a topical antiseptic. It has a broad spectrum of antimicrobial activity against a wide range of bacteria, viruses, and fungi, including yeasts and dermatophytes. TTO is a mixture of more than 100 different compounds, primarily terpenes (mainly monoterpenes and sesquiterpenes). The physical properties and chemical composition of TTO are variable, and it is, therefore, important to determine international standards. The Australian Standard for tea tree oil (AS 2782-1985) includes directives relating to the levels of two components: the minimum content of terpinen-4-ol should be at least 30% and the maximum content of 1,8-cineole should be less than 15% of the oil volume [1]. The international standard for tea tree oil (ISO 4730:2004) includes maximum and minimum percentage values for the 15 most important TTO components. TTO obtained by steam distillation of the leaves and terminal branches of* Melaleuca alternifolia* Cheel,* Melaleuca linariifolia* Smith,* Melaleuca dissitiflora* F. Mueller, and other species of* Melaleuca* should conform to this standard [2].

TTO has been used for centuries in Australian folk medicine, predominantly for wound treatment [3, 4]. In the 1920s, Penfold described for the first time the properties and chemical composition of TTO, and he later confirmed the antiseptic properties of TTO and its components [5–8]. In the 1930s, consecutive publications appeared which demonstrated the powerful antimicrobial activity of TTO when used in inhalation therapy, aseptic surgery, dental surgery, wound disinfection, and oral cavity rinsing [9–11].

Currently, TTO is used as a local agent for treating various diseases, predominantly dermatoses (e.g., recurrent herpes labialis, acne, pustules, dandruff, and rash). TTO is also used to treat* Staphylococcus aureus* infections of the oral cavity and the pharynx, vaginitis, and respiratory tract diseases. Numerous studies have confirmed the broad antimicrobial activity of TTO against bacteria, fungi, and viruses, as well as microorganisms that are resistant to conventional drugs [12–16]. This is important due to the increase in infections that are difficult to treat, as TTO can be used as an alternative to or in combination with conventional drugs (including antibiotics and chemotherapeutic agents).

Treatment of infections can be based on monotherapy (using one antimicrobial drug) or combined therapy (two or more drugs). The primary aim of combined therapy is to enhance the action of the drugs while decreasing the dosages, through synergism. When monotherapy or combined therapy based on conventional drugs is unsuccessful, then combined treatment including a natural agent may be more effective. Several recent studies have reported the increased antimicrobial activity of natural substances combined with conventional drugs as compared to conventional drug treatment alone [17–20].

The aim of this study was to evaluate the activity of fluconazole against clinical strains of fluconazole-resistant* Candida albicans* and reference strain* C. albicans* ATCC 10231, after their exposure to sublethal concentrations of TTO or its main bioactive component terpinen-4-ol.

## 2. Materials and Methods

### 2.1. *Candida albicans* Strains

This study included 32 clinical* Candida albicans* strains, which were isolated from the following materials: swabs of the pharynx and oral cavity (*n* = 5), vagina (*n* = 15), sputum (*n* = 8), or faeces (*n* = 4). These strains were isolated from culture on Sabouraud agar (bioMèrieux, Marcy l'Etoile, France), and species identification was performed using the biochemical test ID 32C (bioMèrieux, Marcy l'Etoile, France). We also used the reference strain* C. albicans* ATCC 10231, which was purchased from Oxoid Ltd. (Basingstoke, Great Britain). We previously determined the sensitivity of* C. albicans* strains to fluconazole by the Kirby-Bauer disk diffusion susceptibility test [21] using 6 mm filter paper disks impregnated with 10 *μ*g of fluconazole obtained from DHN (Cracow, Poland) and YNB agar (Yeast Nitrogen Base-Difco 0.5%, glucose 3%, agar 1.8%, pH = 7) also obtained from DHN (Cracow, Poland). The* C. albicans* strains were classified as exhibiting susceptibility (diameter of growth inhibition zone ≥18 mm), intermediate susceptibility (diameter of growth inhibition zone from 14 mm to 17 mm), or resistance (diameter of growth inhibition zone <14 mm) to fluconazole (the data were described in chapter 3). The fluconazole MIC (minimal inhibitory concentration) and MFC (minimal fungicidal concentration) values were determined by the broth dilution method according to the Clinical and Laboratory Standards Institute (CLSI document M27-A3-2008) [22]. Using this standard, the* C. albicans* strains were classified as exhibiting susceptibility (MIC ≤ 8 *μ*g/mL), intermediate susceptibility (MIC from 9 *μ*g/mL to 63 *μ*g/mL), or resistance (MIC ≥ 64 *μ*g/mL) to fluconazole (the data were presented in chapter 3).

### 2.2. Tea Tree Oil (TTO)

In this study, we used Australian tea tree oil (*Melaleuca alternifolia*) from Thursday Plantation (Integria Healthcare, Eight Mile Plains, QLD, Australia) series 270930 that conforms to the ISO standard 4730:2004 [2] (Table 1). TTO was distilled from specially selected* Melaleuca alternifolia* leaves, a plant native to the coastal regions of northern New South Wales and south eastern Queensland in Australia. The analysis of TTO composition was carried out by gas chromatography according to the international standard ISO 4730 [2]. It was performed in the following conditions: fused-silica column (50 m × 0,20 mm i.d., film thickness 0,25 *μ*m) and flame ionisation type of detector were used, the carrier gas was hydrogen (flow rate of 1 mL/min), the oven temperature programme was from 70°C to 220°C at a rate of 2°C/min, the injector temperature was 230°C, the detector temperature was 250°C, the volume of injected TTO was 0,2 *μ*L, and the split ratio was 1 : 100.

In our study, we also used terpinen-4-ol, which was obtained from Sigma-Aldrich (St. Louis, MO, USA).

### 2.3. Fluconazole

**Figure 1:**
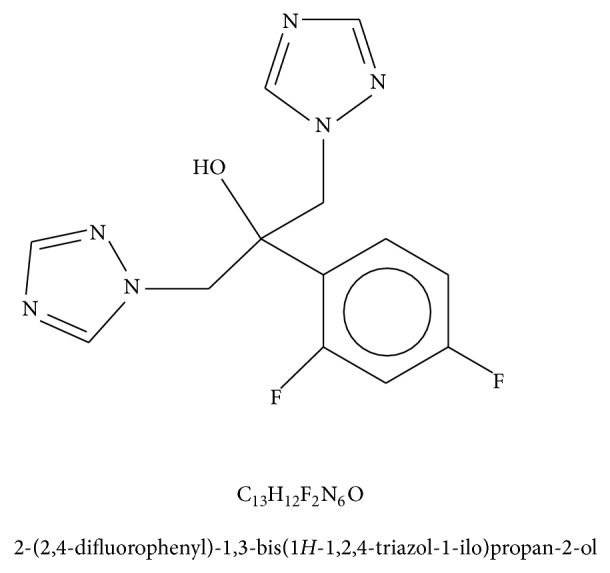
Chemical structure of fluconazole [23].

In this study, we used the antifungal drug fluconazole (Polfarmex, Kutno, Poland). The structure of the fluconazole molecule is shown in Figure 1.

### 2.4. Preparation of the Initial* Candida albicans* Suspension

*C. albicans* cells cultured for 24 h on Sabouraud agar were suspended in a saline solution (0.85% NaCl) and adjusted to a 0,5 McFarland density standard (1,5 × 10^8^ CFU/mL). This suspension was later diluted to a density of 6 × 10^4^ CFU/mL. The suspension was then used to estimate the MIC and MFC values for TTO, terpinen-4-ol, and fluconazole.

### 2.5. Determination of MIC and MFC Values for TTO and Terpinen-4-ol

The TTO activity against the* C. albicans* strains tested was determined by broth macrodilution using the general dilution standards as described by PN-EN ISO 20776-1:2007 [24]. TTO was serially diluted in liquid Sabouraud medium with 10% Tween 80 to final TTO concentrations of 1% to 0.0075%. The Tween 80 detergent helps dissolve the TTO. The same volume of the* C. albicans* suspension was added to each tube to obtain a final density of 3 × 10^3^  CFU/mL. After 24 h of incubation at 35°C, the cell growth was assessed visually in the tubes with TTO and the positive control tube (without TTO). The MIC was defined as the lowest concentration of TTO that led to no visible growth of the cell strains tested. The MFC value was defined as the lowest concentration of TTO that showed no growth of* C. albicans* colonies. The experiment was performed triply. The terpinen-4-ol MIC and MFC values were determined identically as described above. The TTO and terpinen-4-ol MICs were used to calculate the sublethal doses of TTO and terpinen-4-ol used in the following experiments.

### 2.6. Brief Pretreatment of* Candida albicans* with 1/4 MIC TTO

For each sample, a tube was prepared containing saline solution with 10% Tween 80 and TTO to a final concentration of 1/4 MIC TTO. A control tube with no TTO was also prepared. Next, the* C. albicans* suspension was added to tubes to obtain a final density of 3 × 10^3^ CFU/mL. The suspensions were then incubated at 35°C for 30 minutes. The samples were then rinsed twice and centrifuged between rinses (3000 ×g, 15 minutes), and the cells were resuspended to a density of 6 × 10^4^ CFU/mL. The suspension was then used to determine the fluconazole MIC and the minimal fungicidal concentration (MFC) of fluconazole. The study was performed in triplicate.

### 2.7. Determination of the Fluconazole MIC and MFC Values after Brief Pretreatment of* Candida albicans* with 1/4 MIC TTO

The fluconazole activity against the* C. albicans* strains tested was determined by broth macrodilution using the general dilution standards as described by PN-EN ISO 20776-1:2007 [24]. Serial, parallel dilutions of fluconazole ranging from 256.0 *μ*g/mL to 0.125 *μ*g/mL were prepared in liquid Sabouraud medium, and a control tube without the drug was included. For each of the tubes, the same volume of* C. albicans* cells suspension pretreated with 1/4 MIC TTO was added, and the inoculum was adjusted to a final density of 3 × 10^3^CFU/mL. After 24 h of incubation at 35°C, the cell growth in each tube was assessed visually. The MIC value was defined as the lowest concentration of fluconazole that resulted in no visible growth of the strains tested. The cells from the tube identified as the MIC, as well as several of the surrounding dilutions, were plated to Sabouraud agar. After 24 h of incubation at 35°C, the* C. albicans* colonies were counted. The MFC value was defined as the lowest concentration of fluconazole that showed no growth of* C. albicans* colonies. The experiment was performed in triplicate. The* C. albicans* strains were classified as exhibiting susceptibility, intermediate susceptibility, or resistance to fluconazole according to CLSI document M27-A3-2008 [22], as described in Section 2.1.

### 2.8. Prolonged Pretreatment of* Candida albicans* with Fluconazole and Sublethal Dose of TTO or Terpinen-4-ol

Serial, parallel dilutions of fluconazole ranging from 256.0 *μ*g/mL to 0.125 *μ*g/mL were prepared in liquid Sabouraud culture medium. Two positive controls were included. All tubes contained 10% Tween 80, and TTO was added to each dilution and one of the control tubes to achieve a final concentration of 1/4 MIC TTO. The second control tube contained only the liquid medium. Next, an equal volume of* C. albicans* suspension was added to each tube to a final density of 3 × 10^3^ CFU/mL. All the tubes were incubated at 35°C for 24 h. After incubation, the cell growth in each tube was evaluated visually, and the fluconazole MIC and MFC values were defined, as described previously. The cells from the tube identified as the MIC, as well as several of the surrounding dilutions, were plated to Sabouraud agar. After 24 h of incubation at 35°C, the* C. albicans* colonies were counted, and the fluconazole MFC value was defined. The experiment was performed in triplicate. The prolonged pretreatment of* C. albicans* with fluconazole and terpinen-4-ol was performed identically as described above.

### 2.9. Statistical Methods

The results are presented as the arithmetic mean and the median. The statistical differences between the mean values were determined by Student's *t*-test and the Mann-Whitney *U* test, depending on how well the results correlated with a normal distribution. Values of *P* ≤ 0.05 were considered statistically significant. The programme STATISTICA version 10 (StatSoft, Cracow, Poland) was used to perform the statistical analyses.

## 3. Results

The* Candida albicans* strains tested were resistant to fluconazole and susceptible to low concentrations of TTO. The clinical* C. albicans* strains and* C. albicans* ATCC 10231 reference strain, tested by the Kirby-Bauer disk diffusion susceptibility test, did not exhibit the zone of inhibition of growth. All the studied* C. albicans* strains were classified as exhibiting resistance to fluconazole. The fluconazole MIC values for the 32 clinical* C. albicans* strains ranged from 64.0 *μ*g/mL to 256.0 *μ*g/mL (average = 244.0 ± 47.22 *μ*g/mL). The most common values were 256.0 *μ*g/mL (30 strains) and 64.0 *μ*g/mL (2 strains). For* C. albicans* ATCC 10231 reference strain the fluconazole MIC was 256.0 *μ*g/mL.

The TTO MICs for the 32 clinical* C. albicans* strains ranged from 0.06% to 0.5% (average = 0.19 ± 0.09%). The most common values were 0.125% (15 strains) and 0.25% (15 strains). The TTO MICs of the two remaining strains were 0.06% and 0.5%. For* C. albicans* ATCC 10231 reference strain, the TTO MIC was 0.125%. These results indicate that the* C. albicans* strains tested did not exhibit any cross-resistance to TTO and fluconazole. The TTO MIC values were used to calculate the sublethal doses (1/4 MIC TTO) used in the rest of the study.

The brief pretreatment of 32 clinical* C. albicans* strains and of* C. albicans* ATCC 10231 reference strain with 1/4 MIC TTO did not change the fluconazole MIC and MFC values. Exposing* C. albicans* strains to 1/4 MIC TTO and fluconazole for 24 hours (prolonged pretreatment) significantly increased susceptibility yeast strains to fluconazole. Out of 32 fluconazole-resistant* C. albicans* clinical strains, 28 strains (87.5%) exhibited then high or intermediate susceptibility to fluconazole (Table 2).

Exposure of fluconazole-resistant* C. albicans* strains for 24 h to 1/4 MIC TTO and fluconazole enhanced fluconazole activity against these strains. Overall, 62.5% of isolates were classified as susceptible, 25.0% exhibited intermediate susceptibility, and 12.5% were resistant. For all of the tested clinical strains, the average fluconazole MIC decreased from 244.0 *μ*g/mL to 38.46 *μ*g/mL after this prolonged pretreatment, and the average fluconazole MFC decreased from 254.67 *μ*g/mL to 66.62 *μ*g/mL (Table 3). The MIC and MFC values for the susceptible strains (*n* = 20) and strains with intermediate susceptibility (*n* = 8) were statistically low compared to the analogous values obtained for the control sample and for the samples that were only briefly pretreated with TTO. For the group of susceptible isolates, the fluconazole MIC decreased to an average of 0.52 *μ*g/mL, and the fluconazole MFC decreased to an average of 4.25 *μ*g/mL. Prolonged pretreatment of* Candida albicans* ATCC 10231 standard strain with 1/4 MIC TTO and fluconazole did not increase the susceptibility of this strain to fluconazole, like the four fluconazole-resistant clinical* C. albicans* strains studied.

Terpinen-4-ol, the main bioactive component present in TTO, strongly enhanced fluconazole activity against fluconazole-resistant* C. albicans* strains. The terpinen-4-ol MICs for clinical* C. albicans* strains ranged from 0.06% to 0.25% (average = 0.11 ± 0.09%). For* C. albicans* ATCC 10231 standard strain, the terpinen-4-ol MIC was 0.06%. The* C. albicans* strains tested did not exhibit any cross-resistance to terpinen-4-ol and fluconazole. Exposure of fluconazole-resistant clinical and standard* C. albicans* strains for 24 h to fluconazole and sublethal doses (1/4 MIC) of terpinen-4-ol strongly enhanced fluconazole activity against these strains, and all of* C. albicans* isolates were classified as susceptible (fluconazole MIC decreased to 0.125 *μ*g/mL). We summed up the results of this study, and the most important data are presented in a table form (Table 4).

## 4. Discussion

TTO is the most commonly used essential oil for its antibacterial and antifungal properties [3, 25]. In this study, we evaluated the change in fluconazole activity* in vitro* against fluconazole-resistant clinical* Candida albicans* strains exposed to the sublethal concentrations of TTO or terpinen-4-ol, the main bioactive component of TTO. The earlier* in vitro* studies of the sensitivity of* Candida* spp. to TTO have shown that TTO is highly active against these microbes, as well as azole-resistant strains, for which the TTO MICs ranged from 0.25% to 0.5% [14, 26]. For the* C. albicans* strains that were resistant to both fluconazole and itraconazole, the TTO MICs ranged from 0.25 to 1.0%, the TTO MIC_50_ was 0.5%, and the TTO MIC_90_ was 1% [27]. Another study showed that three fluconazole-resistant clinical* C. albicans* strains had very low TTO MICs (0.15% for two strains and 0.07% for the third strain) [15].

The experiments performed in this study confirm the results from previously published studies in that all of the tested fluconazole-resistant* C. albicans* strains were sensitive to TTO [14, 15, 28, 29]. The determined TTO MICs were low, ranging from 0.06% to 0.5%. The TTO antimicrobial activity is attributed mainly to terpinen-4-ol, the main bioactive component present in TTO [3, 14]. The determined MIC values for terpinen-4-ol were very low, ranging from 0.06% to 0.25%. Our study and other studies show that* C. albicans* does not exhibit cross-resistance to TTO and azole agents [14, 15, 26, 27]. Clinical resistance to TTO has not been reported. Multicomponent nature of TTO may reduce the potential for resistance to occur spontaneously, and multiple simultaneous mutations may be required to overcome all of the antimicrobial actions of each of the components [3]. Thus, TTO can be used as a topical antiseptic to effectively treat superficial mycoses caused by fluconazole-resistant* Candida* spp. and other azole-resistant yeast. Unfortunately, TTO can be potentially toxic when it is ingested in high doses, and, therefore, TTO should not be administrated orally. The acute oral toxicity of TTO is similar to the oral toxicity of other common essential oils, for example, such as eucalyptus oil [30, 31]. The lipophilic nature of TTO, which enables it to penetrate the outer layers of skin, potentiates not only the antiseptic actions but also the possibility of TTO toxicity due to dermal absorption. TTO can cause skin irritation at higher concentrations and may cause allergic reactions in predisposed individuals [3, 31, 32]. Zhang and Robertson observed ototoxic effect of 100% TTO [33]. The toxicity of TTO is dose-dependent, and the majority of adverse events can be avoided through the use of TTO in a diluted form [31]. TTO is not mutagenic or genotoxic [34, 35].

There is increasing interest not only in the activity of natural substances against resistant microbes but also in the synergistic interactions between these substances and conventional drugs [19, 20, 36–38]. Fluconazole is one of the azole antifungal agents widely used for both prophylaxis and therapy of* Candida* infections [39–41]. In this study, we explored changes in the activity of fluconazole against fluconazole-resistant* C. albicans* strains after exposure to sublethal concentrations of TTO or terpinen-4-ol. We used exclusively fluconazole-resistant strains because identifying synergistic treatments for these strains would be especially important. We tested sublethal concentrations of TTO and terpinen-4-ol because we expected concentrations lower than the MIC to weaken the cell structure without killing the cells, facilitating the activity of fluconazole and consequently inhibiting* C. albicans* resistance to fluconazole. Our results show that brief (0.5 h) exposure of fluconazole-resistant* C. albicans* strains to sublethal concentration of TTO (1/4 MIC TTO) had no influence on the antifungal activity of fluconazole. However, exposing* C. albicans* cells to sublethal concentration of TTO and then treating them with fluconazole inhibited the resistance to fluconazole in 87.5% of the tested strains. These results suggest that there is a synergistic interaction between fluconazole and TTO against fluconazole-resistant* C. albicans*. TTO was used to permeabilise the yeast cell membranes, markedly increasing the susceptibility to fluconazole. The TTO becomes embedded in the lipid bilayer membrane, which disrupts its structure, resulting in increased permeability and impaired physiological function. TTO also inhibits the formation of germ tubes or mycelial conversion in* C. albicans* and inhibits respiration in* C. albicans* in dose-dependent manner [3]. Fungal cells exposed to TTO will eventually rupture. Sublethal concentrations of TTO also weaken* Candida* spp. cells vitality [41, 42]. The mechanism of fluconazole antifungal activity is different. It was demonstrated that fluconazole interferes with the cytochrome P-450-dependent enzyme C-14*α*-demethylase, which is responsible for production of ergosterol. The disruption of ergosterol synthesis causes structural and functional changes in the fungal cell membrane, which predispose the fungus cells to damage. Inhibition of cytochrome *c* oxidative and peroxidative enzymes is an additional antifungal activity of fluconazole [39]. Several mechanisms have been described for fluconazole resistance in* C. albicans* isolates: increased production of lanosterol 14*α*-demethylase encoded by* ERG11* gene and decreases in the affinity of lanosterol 14*α*-demethylase for fluconazole because of mutations in* ERG11* gene and a defect in Δ5-6 desaturase encoded by* ERG3* gene causing loss of function in the ergosterol pathway. The other mechanism of fluconazole resistance in* C. albicans* is the active transport of drugs across the plasma membrane by “efflux pumps,” which requires the expression of the* CDR1/2* and* MDR1* genes [39, 43–47]. TTO-induced cell membrane damage can disrupt the function of “efflux pumps,” thus making the fungal cell more susceptible to fluconazole [48, 49].

Our data show that there is a synergistic effect* in vitro* of sublethal concentrations of TTO and fluconazole against fluconazole-resistant* C. albicans* strains. However, the fluconazole-resistant* C. albicans* ATCC 10231 standard strain and four clinical* C. albicans* strains did not increase the susceptibility to fluconazole. The differences in mechanisms of resistance of these strains to fluconazole were probable cause of this effect. In our* in vitro* study the TTO main component terpinen-4-ol was more active than TTO and strongly enhanced fluconazole activity against all studied fluconazole-resistant* C. albicans* strains. Mondello et al. [14] as well as Ninomiya et al. [50] observed that* in vivo* TTO and terpinen-4-ol were similarly effective against candidiasis caused by azole-resistant* C. albicans*. The mechanisms underlying the synergy between fluconazole and TTO did not elucidate. Yu et al. [51] confirmed the synergism between fluconazole and triclosan against clinical isolates of fluconazole-resistant* C. albicans*. Liu et al. [52] observed synergistic effect between fluconazole and glabridin against* C. albicans* related to the effect of glabridin on cell envelope. Ahmad et al. [53] described synergistic activity of thymol and carvacrol with fluconazole against* Candida* isolates. Both monoterpenes inhibited efflux by 70–90% showing their high potency to block drug transporter pumps.

Previous studies also have evaluated the activity of TTO against various microorganisms in combination with other antimicrobial substances. A synergistic effect was observed for itraconazole and TTO in a thermosensitive gel used to treat vaginal candidiasis [26]. Synergistic effects have also been observed between essential oils and ciprofloxacin, gentamicin, cefixime, and pristinamycin [20]. In a disc diffusion test using* C. albicans, C. glabrata, C. tropicalis, C. krusei, C. guilliermondii*, and* C. parapsilosis*, larger growth inhibition zones occurred around discs impregnated with TTO and amphotericin B than around discs containing only TTO [17]. In a study of* Staphylococcus aureus*, larger zones of growth inhibition occurred around discs impregnated with TTO and other essential oils compared to discs impregnated with TTO only [54].

The synergistic action of antimicrobial substances has also been shown using* time-kill* curves. The short pretreatment of* Pseudomonas aeruginosa* with a substance that disrupts the cytoplasmic membrane (carbonyl cyanide m-chlorophenylhydrazone, polymyxin B nonapeptide, or ethylenediaminetetraacetic acid) enhanced the bactericidal activity of TTO, as demonstrated by the increased speed of microbe killing in the* time-kill* curves [55, 56]. However, in a study using the E-test method,* Escherichia coli, Salmonella enteritidis, Salmonella typhimurium, Staphylococcus aureus*, and coagulase-negative staphylococci (CoNS) exposed to sublethal concentrations of TTO for 72 hours exhibited increased resistance to gentamicin, streptomycin, chloramphenicol, tetracycline, erythromycin, trimethoprim, ampicillin, fusidic acid, mupirocin, linezolid, and vancomycin [57, 58]. Increased antimicrobial activity was observed when essential oils were combined with their isolated components (e.g., terpinen-4-ol from* Melaleuca alternifolia*) [59] and when TTO was combined with silver ions [60, 61].

The fractional inhibition concentration (FIC) index, also referred to as the FICI, is used to determine whether two substances are synergistic or antagonistic. FIC values can be interpreted differently, however, in general, an FIC index lower than 0.5 indicates synergism and an FIC index higher than 4 indicates antagonism [18, 19, 38, 59]. The FIC index value for TTO and tobramycin was 0.37 for* Escherichia coli* and 0.62 for* Staphylococcus aureus*, indicating that these two substances are synergistic [19]. A minor synergistic effect was observed when treating* Candida albicans* with TTO and amphotericin B and* Klebsiella pneumoniae* with TTO and ciprofloxacin. TTO and ciprofloxacin exhibit antagonistic effects against* Staphylococcus aureus* [18]. There is no synergistic effect between TTO and lysostaphin, mupirocin, gentamicin, or vancomycin against methicillin-resistant* Staphylococcus aureus* strains. In fact, the FIC index indicated that TTO and vancomycin are antagonistic [38].

The results of this study and other previous studies demonstrate that combining natural substances such as TTO and conventional drugs such as fluconazole may help treat difficult yeast infections. However, additional* in vitro* studies are needed to identify the antimicrobial activity of natural medicinal substances and detect synergistic interactions with commonly used antimicrobial agents.

## Figures and Tables

**Table 1:** Composition of the TTO used in this study compared to ISO standard 4730:2004 [2].

Components	Content (%) according to ISO standard 4730	Content (%) of TTO sample
*α*-Pinene	1–6	2.5
Sabinene	Trace-3.5	0.1
*α*-Terpinene	5–13	8.1
Limonene	0.5–1.5	1.0
*p*-Cymene	0.5–8	4.4
1,8-Cineole	Trace-15	2.8
*γ*-Terpinene	10–28	19.6
Terpinolene	1.5–5	3.2
Terpinen-4-ol	30–48	41.0
*α*-Terpineol	1.5–8	3.0
Aromadendrene	Trace-3	1.3
Ledene (syn. viridiflorene)	Trace-3	No data available
*δ*-Cadinene	Trace-3	No data available
Globulol	Trace-1	No data available
Viridiflorol	Trace-1	No data available

**Table 2:** Susceptibility of fluconazole-resistant clinical *Candida albicans* strains to fluconazole after exposure to 1/4 MIC TTO.

*Candida albicans* strains (*n* = 32)	Number (%) of *Candida albicans* strains with the indicated susceptibility to fluconazole		
	Resistant	Intermediate susceptibility	Susceptible
Strains not exposed to TTO (control)	32 (100%)	0	0
Strains exposed to 1/4 MIC TTO for 30 minutes	32 (100%)	0	0
Strains exposed to 1/4 MIC TTO and fluconazole for 24 hours	4 (12.5%)	8 (25.0%)	20 (62.5%)

**Table:** (a) Fluconazole MIC values (*μ*g/mL)

	*C. albicans* (*n* = 32)^a^			*C. albicans* (*n* = 20)^b^			*C. albicans* (*n* = 8)^c^		
	Control	Brief pretreatment with TTO	Prolonged pretreatment with TTO and fluconazole	Control	Brief pretreatment with TTO	Prolonged pretreatment with TTO and fluconazole	Control	Brief pretreatment with TTO	Prolonged pretreatment with TTO and fluconazole
Range of MICs	64.0–256.0	64.0–256.0	0.125–256.0	256.0-256.0	256.0-256.0	0.125–2.67	64.0–256.0	64.0–256.0	12.0–42.67
Average MIC	244.0 ± 47.22	244.0 ± 47.22	38.46 ± 84.35	256.0 ± 0.0	256.0 ± 0.0	0.52 ± 0.56	208.0 ± 88.88	208.0 ± 88.88	24.54 ± 11.54
*P* ^d^			*P* < 0.0001^e^ *P* < 0.0001^f^			*P* < 0.0001^e^ *P* < 0.0001^f^			*P* < 0.0002^e^ *P* < 0.0002^f^

**Table:** (b) Fluconazole MFC values (*μ*g/mL)

	*C. albicans* (*n* = 32)^a^			*C. albicans* (*n* = 20)^b^			*C. albicans* (*n* = 8)^c^		
	Control	Brief pretreatment with TTO	Prolonged pretreatment with TTO and fluconazole	Control	Brief pretreatment with TTO	Prolonged pretreatment with TTO and fluconazole	Control	Brief pretreatment with TTO	Prolonged pretreatment with TTO and fluconazole
Range of MFCs	213.33–256.0	256.0-256.0	0.17–256.0	256.0-256.0	256.0-256.0	0.17–23.33	213.33–256.0	256.0-256.0	14.67–213.33
Average MFC	254.48 ± 7.54	256.0 ± 0.0	66.62 ± 96.16	256.0 ± 0.0	256.0 ± 0.0	4.25 ± 6.19	250.67 ± 15.08	256.0 ± 0.0	127.83 ± 70.42
*P* ^d^			*P* < 0.0001^e^ *P* < 0.0001^f^			*P* < 0.0001^e^ *P* < 0.0001^f^			*P* < 0.0003^e^ *P* < 0.0002^f^

^a^All 32 tested fluconazole-resistant clinical *Candida albicans* strains.

^b^Fluconazole-resistant clinical *Candida albicans* strains (*n* = 20) that exhibited susceptibility to fluconazole after prolonged pretreatment with TTO.

^c^Fluconazole-resistant clinical *Candida albicans* strains (*n* = 8) that exhibited intermediate susceptibility to fluconazole after prolonged pretreatment with TTO.

*P* ^d^: the level of statistical significance for the average MIC/MFC values.

*P* ^e^: statistical significance compared to the control.

*P* ^f^: statistical significance compared to the group that was pretreated briefly.

**Table 4:** MIC and MFC values of fluconazole, TTO, terpinen-4-ol, and fluconazole with TTO or terpinen-4-ol, for fluconazole-resistant *Candida albicans* strains.

Reagents	*C. albicans* ATCC 10231		*C. albicans* clinical strains (*n* = 32)			
	MIC	MFC	MIC		MFC	
			Range	Average	Range	Average
Fluconazole (*μ*g/mL)	256.0	256.0	64.0–256.0	244.0 ± 47.22	213.33–256.0	254.48 ± 7.44
TTO (% v/v)	0.125	0.25	0.06–0.5	0.19 ± 0.09	0.125–0.5	0.37 ± 0.13
Fluconazole (*μ*g/mL) with sublethal dose of TTO	256.0	256.0	0.125–256.0	38.46 ± 84.35	0.17–256.0	66.62 ± 96.16
Terpinen-4-ol (% v/v)	0.06	0.125	0.06–0.25	0.11 ± 0.09	0.125–0.5	0.22 ± 0.19
Fluconazole (*μ*g/mL) with sublethal dose of terpinen-4-ol	0.125	0.125	0.125-0.125	0.125 ± 0.0	0.125–1.0	0.38 ± 0.42

## References

[1] Standards Association of Australia (1985). *AS 2782:1985, Essential Oils-Oil of Melaleuca, Terpinen-4-ol Type*.

[2] International Organisation for Standardization (2004). Oil of Melaleuca, terpinen-4-ol type (Tea Tree oil). *International Standard ISO*.

[3] Carson C. F., Hammer K. A., Riley T. V. (2006). Melaleuca alternifolia (tea tree) oil: a review of antimicrobial and other medicinal properties. *Clinical Microbiology Reviews*.

[4] Wilkinson J. M., Cavanagh H. M. A. (2005). Antibacterial activity of essential oils from Australian native plants. *Phytotherapy Research*.

[5] Penfold A. R. (1925). The essential oils of *Melaleuca linariifolia* (Smith) and *Melaleuca alternifolia* (Cheel). *Journal of the Royal Society of New South Wales*.

[6] Penfold A. R., Grant R. (1925). The germicidal values of some Australian essential oils and their pure constituents. Together with those for some essential oil isolates, and synthetics. Part III. *Journal of the Royal Society of New South Wales*.

[7] Penfold A. R., Grant R. (1923). The germicidal values of the principal commercial Eucalyptus oils and their pure constituents, with observations on the value of concentrated disinfectants. *Journal of the Royal Society of New South Wales*.

[8] Penfold A. R., Grant R. (1922). The economic utilization of the residues from the steam rectification of the essential oil of *Eucalyptus cneorifolia* and the germicidal values of the crude and the pure active constituents. *Journal of the Royal Society of New South Wales*.

[9] (1933). An Australian antiseptic oil. *British Medical Journal*.

[10] Humphery E. M. (1930). A new Australian germicide. *Medical Journal of Australia*.

[11] Anonymous (1933). Ti-troll oil. *British Medical Journal*.

[12] Vazquez J. A., Arganoza M. T., Boikov D., Vaishampayan J. K., Akins R. A. (2000). In vitro susceptibilities of *Candida* and *Aspergillus* species to *Melaleuca alternafolia* (tea tree) oil. *Revista Iberoamericana de Micologia*.

[13] Vasquez J. A., Zawawi A. A. (2002). Efficacy of alcohol-based and alcohol-free *melaleuca* oral solution for the treatment of fluconazole-refractory oropharyngeal candidiasis in patients with AIDS. *HIV Clinical Trials*.

[14] Mondello F., de Bernardis F., Girolamo A., Cassone A., Salvatore G. (2006). *In vivo* activity of terpinen-4-ol, the main bioactive component of *Melaleuca alternifolia* Cheel (tea tree) oil against azole-susceptible and -resistant human pathogenic *Candida* species. *BMC Infectious Diseases*.

[15] Traboulsi R. S., Mukherjee P. K., Ghannoum M. A. (2008). *In vitro* activity of inexpensive topical alternatives against *Candida* spp. isolated from the oral cavity of HIV-infected patients. *International Journal of Antimicrobial Agents*.

[16] Brady A., Loughlin R., Gilpin D., Kearney P., Tunney M. (2006). *In vitro* activity of tea-tree oil against clinical skin isolates of meticillin-resistant and -sensitive *Staphylococcus aureus* and coagulase-negative staphylococci growing planktonically and as biofilms. *Journal of Medical Microbiology*.

[17] Rosato A., Vitali C., Gallo D., Balenzano L., Mallamaci R. (2008). The inhibition of *Candida* species by selected essential oils and their synergism with amphotericin B. *Phytomedicine*.

[18] Van Vuuren S. F., Suliman S., Viljoen A. M. (2009). The antimicrobial activity of four commercial essential oils in combination with conventional antimicrobials. *Letters in Applied Microbiology*.

[19] D'Arrigo M., Ginestra G., Mandalari G., Furneri P. M., Bisignano G. (2010). Synergism and postantibiotic effect of tobramycin and *Melaleuca alternifolia* (tea tree) oil against Staphylococcus aureus and *Escherichia coli*. *Phytomedicine*.

[20] Fadli M., Saad A., Sayadi S. (2012). Antibacterial activity of *Thymus maroccanus* and *Thymus broussonetii* essential oils against nosocomial infection—bacteria and their synergistic potential with antibiotics. *Phytomedicine*.

[21] Bauer A. W., Kirby W. M., Sherris J. C., Turck M. (1966). Antibiotic susceptibility testing by a standardized single disk method. *American Journal of Clinical Pathology*.

[22] National Committee for Clinical Laboratory Standards/Clinical and Laboratory Standards Institute (NCCLS/CLSI) (2008). Reference method for broth dilution antifungal susceptibility testing of yeasts: approved standard—third edition. *CLSI document*.

[23] (2008). Fluconazolum (Flukonazol). *Farmakopea Polska VIII*.

[24] Polski Komitet Normalizacyjny (2007). Kliniczne badania laboratoryjne i metody badań diagnostycznych in vitro. Oznaczanie wrażliwości drobnoustrojów i ocena przydatności gotowych testów do oznaczania wrażliwości na leki przeciwbakteryjne.

[25] Stea S., Beraudi A., De Pasquale D. (2014). Essential oils for complementary treatment of surgical patients: state of the art. *Evidence-Based Complementary and Alternative Medicine*.

[26] Mondello F., de Bernardis F., Girolamo A., Salvatore G., Cassone A. (2003). *In vitro* and *in vivo* activity of tea tree oil against azole-susceptible and -resistant human pathogenic yeasts. *Journal of Antimicrobial Chemotherapy*.

[27] Bagg J., Jackson M. S., Petrina Sweeney M., Ramage G., Davies A. N. (2006). Susceptibility to *Melaleuca alternifolia* (tea tree) oil of yeasts isolated from the mouths of patients with advanced cancer. *Oral Oncology*.

[28] Thosar N., Basak S., Bahadure R. N., Rajurkar M. (2013). Antimicrobial efficacy of five essential oils against oral pathogens: an *in vitro* study. *European Journal of Dentistry*.

[29] Sharma S., Hegde V. (2014). Comparative evaluation of antifungal activity of *Melaleuca* oil and fluconazole when incorporated in tissue conditioner: an in vitro study. *Journal of Prosthodontics*.

[30] Carson C. F., Riley T. V. (1993). Antimicrobial activity of the essential oil of *Melaleuca alternifolia*. *Letters in Applied Microbiology*.

[31] Hammer K. A., Carson C. F., Riley T. V., Nielsen J. B. (2006). A review of the toxicity of *Melaleuca alternifolia* (tea tree) oil. *Food and Chemical Toxicology*.

[32] Rutherford T., Nixon R., Tam M., Tate B. (2007). Allergy to tea tree oil: retrospective review of 41 cases with positive patch tests over 4.5 years. *Australasian Journal of Dermatology*.

[33] Zhang S. Y., Robertson D. (2000). A study of tea tree oil ototoxicity. *Audiology Neuro-Otology*.

[34] Pazyar N., Yaghoobi R., Bagherani N., Kazerouni A. (2013). A review of applications of tea tree oil in dermatology. *International Journal of Dermatology*.

[35] Klimmek J. K. W., Nowicki R., Szendzielorz K. (2002). Application of a tea tree oil and its preparations in combined treatment of dermatomycoses. *Mikologia Lekarska*.

[36] Mirza M. A., Ahmad S., Mallick M. N., Manzoor N., Talegaonkar S., Iqbal Z. (2013). Development of a novel synergistic thermosensitive gel for vaginal candidiasis: an *in vitro*, *in vivo* evaluation. *Colloids and Surfaces B: Biointerfaces*.

[37] LaPlante K. L. (2007). *In vitro* activity of lysostaphin, mupirocin, and tea tree oil against clinical methicillin-resistant *Staphylococcus aureus*. *Diagnostic Microbiology and Infectious Disease*.

[38] Hollier L. M., Cox S. M. (1995). Fluconazole (Diflucan). *Infectious Diseases in Obstetrics and Gynecology*.

[39] Kabir M. A., Ahmad Z. (2013). *Candida* infections and their prevention. *ISRN Preventive Medicine*.

[40] Eksi F., Gayyurhan E. D., Balci I. (2013). *In vitro* susceptibility of *Candida* species to four antifungal agents assessed by the reference broth microdilution method. *The Scientific World Journal*.

[41] Paduch R., Kandefer-Szerszeń M., Trytek M., Fiedurek J. (2007). Terpenes: substances useful in human healthcare. *Archivum Immunologiae et Therapiae Experimentalis*.

[42] Hammer K. A., Carson C. F., Riley T. V. (2004). Antifungal effects of *Melaleuca alternifolia* (tea tree) oil and its components on *Candida albicans* , *Candida glabrata* and *Saccharomyces cerevisiae*. *Journal of Antimicrobial Chemotherapy*.

[43] Cernicka J., Subik J. (2006). Resistance mechanisms in fluconazole-resistant *Candida albicans* isolates from vaginal candidiasis. *International Journal of Antimicrobial Agents*.

[44] Niimi M., Firth N. A., Cannon R. D. (2010). Antifungal drug resistance of oral fungi. *Odontology*.

[45] Hiller D., Stahl S., Morschhäuser J. (2006). Multiple *cis*-acting sequences mediate upregulation of the *MDR1* efflux pump in a fluconazole-resistant clinical *Candida albicans* isolate. *Antimicrobial Agents and Chemotherapy*.

[46] Manastir L., Ergon M. C., Yücesoy M. (2011). Investigation of mutations in *ERG11* gene of fluconazole resistant *Candida albicans* isolates from Turkish hospitals. *Mycoses*.

[47] Carvalho V. O., Okay T. S., Melhem M. S. C., Szeszs M. W., del Negro G. M. B. (2013). The new mutation L321F in *Candida albicans ERG11* gene may be associated with fluconazole resistance. *Revista Iberoamericana de Micologia*.

[48] Cox S. D., Mann C. M., Markham J. L. (2000). The mode of antimicrobial action of the essential oil of *Melaleuca alternifolia* (tea tree oil). *Journal of Applied Microbiology*.

[49] Giordani C., Molinari A., Toccacieli L. (2006). Interaction of tea tree oil with model and cellular membranes. *Journal of Medicinal Chemistry*.

[50] Ninomiya K., Maruyama N., Inoue S. (2012). The essential oil of *Melaleuca alternifolia* (tea tree oil) and its main component, terpinen-4-ol protect mice from experimental oral candidiasis. *Biological and Pharmaceutical Bulletin*.

[51] Yu L., Ling G., Deng X., Jin J., Jin Q., Guo N. (2011). *In vitro* interaction between fluconazole and triclosan against clinical isolates of fluconazole-resistant *Candida albicans* determined by different methods. *Antimicrobial Agents and Chemotherapy*.

[52] Liu W., Li L. P., Zhang J. D. (2014). Synergistic antifungal effect of glabridin and fluconazole. *PLoS ONE*.

[53] Ahmad A., Khan A., Manzoor N. (2013). Reversal of efflux mediated antifungal resistance underlies synergistic activity of two monoterpenes with fluconazole. *European Journal of Pharmaceutical Sciences*.

[54] Edwards-Jones V., Buck R., Shawcross S. G., Dawson M. M., Dunn K. (2004). The effect of essential oils on methicillin-resistant *Staphylococcus aureus* using a dressing model. *Burns*.

[55] Longbottom C. J., Carson C. F., Hammer K. A., Mee B. J., Riley T. V. (2004). Tolerance of *Pseudomonas aeruginosa* to *Melaleuca alternifolia* (tea tree) oil is associated with the outer membrane and energy-dependent cellular processes. *Journal of Antimicrobial Chemotherapy*.

[56] Mann C. M., Cox S. D., Markham J. L. (2000). The outer membrane of *Pseudomonas aeruginosa* NCTC 6749 contributes to its tolerance to the essential oil of *Melaleuca alternifolia* (tea tree oil). *Letters in Applied Microbiology*.

[57] McMahon M. A. S., Blair I. S., Moore J. E., McDowell D. A. (2007). Habituation to sub-lethal concentrations of tea tree oil (*Melaleuca alternifolia*) is associated with reduced susceptibility to antibiotics in human pathogens. *Journal of Antimicrobial Chemotherapy*.

[58] McMahon M. A., Tunney M. M., Moore J. E., Blair I. S., Gilpin D. F., McDowell D. A. (2008). Changes in antibiotic susceptibility in staphylococci habituated to sub-lethal concentrations of tea tree oil (*Melaleuca alternifolia*). *Letters of Applied Microbiology*.

[59] Bassolé I. H. N., Juliani H. R. (2012). Essential oils in combination and their antimicrobial properties. *Molecules*.

[60] Chladek G., Mertas A., Barszczewska-Rybarek I. (2011). Antifungal activity of denture soft lining material modified by silver nanoparticles-a pilot study. *International Journal of Molecular Sciences*.

[61] Low W. L., Martin C., Hill D. J., Kenward M. A. (2013). Antimicrobial efficacy of liposome-encapsulated silver ions and tea tree oil against *Pseudomonas aeruginosa*, *Staphylococcus aureus* and *Candida albicans*. *Letters in Applied Microbiology*.

